# Oblique insertion of a straight cage during single level TLIF procedure proves to be non-inferior in terms of restoring segmental lordosis

**DOI:** 10.1016/j.bas.2021.100302

**Published:** 2021-10-16

**Authors:** Peter Truckenmueller, Marcus Czabanka, Simon H. Bayerl, Robert Mertens, Peter Vajkoczy

**Affiliations:** Department of Neurosurgery, Charité – Universitätsmedizin Berlin, Charitéplatz 1, 10117, Berlin, Germany

**Keywords:** Guidelines, Infection prevention, Questionnaire, Spine surgery, Surgical site infections, Survey

## Abstract

**Introduction:**

With increasing relevance of the postoperative spinopelvic alignment, achieving optimal restoration of segmental lordosis (SL) during transforaminal lumbar interbody fusion (TLIF) has become increasingly important. However, despite the easier insertion of the straight cage, its potential to restore SL is still considered inferior to the preferred insert-and-rotate technique with a banana-shaped cage.

**Research question:**

To determine, if simple oblique insertion of a straight cage allows for an equally effective restoration of SL, but reduces risk for intraoperative cage subsidence requiring revision surgery.

**Material and methods:**

The authors retrospectively identified 81 patients who underwent single-level TLIF between 11/2017-03/2020. 40 patients were included in the straight cage group, 41 patients in the banana cage group. The authors determined pre- and postoperative SL from plain lateral radiographs. Bone density was analyzed on computed tomographs using Hounsfield unit (HU) values.

**Results:**

Both cage types were equally effective in restoring SL. However, 7.3% in the banana cage group, but none in the straight cage group, had to undergo revision surgery due to intraoperative cage subsidence. This was related to reduced bone density with lower HU values.

**Discussion:**

With an extended dorsal release, the straight cage may be equally effective in restoring SL. Since no repositioning is needed after oblique insertion, the straight cage might cause less intraoperative endplate violation.

**Conclusion:**

Provided an adequate surgical technique, both cage types might be equally effective in restoring SL after one-level TLIF surgery. However, the straight cage might represent the safer alternative in patients with reduced bone quality.

## Highlights

• Straight cage in single-level TLIF equally effective in restoring segmental lordosis.
• Straight cage might cause less revisions due to intraoperative cage subsidence.
• Straight cage might be safer in patients with reduced bone quality.

## Graphical abstract

**Figure:**
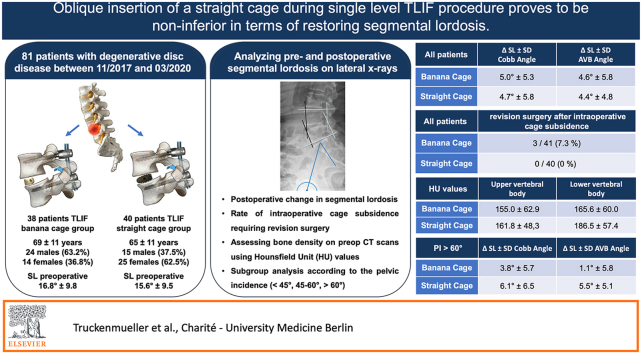

## 1 Introduction

Transforaminal lumbar interbody fusion (TLIF) developed into the preferred procedure for lumbar spinal fusion surgery in degenerative disc disease. Its goal is to achieve a safe 360° fusion with reduced pain and nerve irritation. However, with growing recognition of the importance of the spinopelvic alignment, the demands on the TLIF procedure not only include a stable 360° fusion. The interbody cage must also be capable to restore the disc height in order to allow for a sufficient restoration of the segmental lordosis (SL) with a balanced relationship of the lumbar lordosis (LL) and pelvic incidence (PI) (Lovecchio et al., 2020; Ames et al., 2012; Gottfried et al., 2009). The PI is an individually constant anatomical parameter which defines the pelvic morphotype and lumbar alignment (Roussouly et al., 2003, 2005; Schwab et al., 2009) and the relationship between the PI and the LL as well as their surgical implication can be expressed by PI minus LL (PI-LL) (Ames et al., 2012; Schwab et al., 2013). While the degenerative spinal disease causes a loss in LL, compensatory retroversion of the pelvis with increased pelvic tilt (PT) and reduced sacral slope (SS) as well as reduction of thoracic kyphosis finally results in a progressive spinopelvic misalignment with increased back pain and reduced quality of life reflected in worsening HRQOL scores (Barrey et al., 2013; Lafage et al., 2009; Jackson and McManus, 1994). Thus, it is well acknowledged, that PT and the PI-LL mismatch strongly correlate with disability and provide guidance for preoperative patient-specific planning (Schwab et al., 2010, 2013). Further, multiple studies demonstrated the implication of the PI-LL mismatch on the risk assessment of postoperative failure with development of adjacent level disease (ALD) after lumbar spinal fusion surgery (Schwab et al., 2013; Rothenfluh et al., 2015; Aoki et al., 2015; Tempel et al., 2017). The strong influence of the PI, PT and the PI-LL mismatch on the patient's disability and surgical success highlights the importance of restoring SL after TLIF surgery in order to preserve the spinopelvic alignment with a suitable inclination of the pelvis (Jackson and McManus, 1994; Schwab et al., 2010). However, while an effective restoration of the SL mainly depends on patient specific characteristics like preoperative radiographic parameters as well as on technical aspects, the role of the implanted cage and its design in particular still remains controversial.

For most effective restoration of the SL in TLIF surgery, it has repeatedly been proposed to place the interbody cage as anteriorly as possible (Ould-Slimane et al., 2012; Kuhta and Bošnjak, 2019). Cage placement in the anterior column is supposed to provide for a better load share in combination with posterior compression resulting in a greater SL (Kuhta and Bošnjak, 2019; Rice et al., 2016; Cho et al., 2017). Currently, the use of semilunar insert-and-rotate spacers (banana cage) with placement at the anterior apophyseal rim of the vertebral body is widely acknowledged as the preferred procedure (Sears, 2005; Robertson et al., 2018a). This approach demonstrates favorable biochemical characteristics and several studies using this cage have frequently reported a gain in SL about 5–6° (Tye et al., 2016; Galla et al., 2018). However, insertion and rotation of the banana cage can be challenging in patients with impeded anatomical conditions and reduced bone density (Formby et al., 2016; Faundez et al., 2008).

With growing popularity of the minimally invasive TLIF, oblique insertion of a straight cage across the intervertebral space experienced a renaissance (Choi et al., 2018). Inserting a straight cage is not only technically easier but can also be done fully navigated allowing for more accuracy without the need for fluoroscopy (Navarro-Ramirez et al., 2017). It also provides a crucial benefit in the MIS-TLIF featuring a narrower working space (Choi et al., 2018). Most importantly, the straight cage does not require repositioning after oblique insertion - in contrast to the banana cage. This seems to be relevant especially in patients with reduced bone quality. In those patients, aggressive endplate reaming and rotating of the banana cage might entail a higher risk for endplate violation and intraoperative cage subsidence (Formby et al., 2016; Yao et al., 2020). However, despite its technical advantages, the most frequent point of criticism still is, that oblique insertion of the straight cage does not allow for the same restoration of the SL compared to the banana cage (Ould-Slimane et al., 2012; Rice et al., 2016).

In the following, we tested the hypothesis, that performing the TLIF procedure with oblique insertion of a straight cage across the intervertebral disc space allows for the same restoration of the SL as compared to the conventional, cantilever insert-and-rotate technique with the banana cage. In parallel, we assessed the rate of intraoperative cage subsidence requiring revision surgeries and determined the bone quality of the fused segment using HU values on preoperative spinal CT scans. Furthermore, since the amount of LL necessary to maintain a balanced spinopelvic alignment is directly controlled by and proportional to the PI, a high PI may necessitate a higher LL and should be taken into account during appropriate preoperative planning (Mehta et al., 2015). Therefore, in order to study whether oblique insertion of a straight cage allows for an equal restoration of the SL in patients with a high PI, we stratified the patients according to their PI.

## 2 Materials and methods

### 2.1 Patient cohort

In this retrospective cohort study, we included a total of 81 patients who underwent a single-level TLIF surgery in the segments L1-L5 due to a monosegmental degenerative lumbar disc disease, neuroforaminal stenosis or a spondylolisthesis Meyerding I-II. Retrospective data acquisition was authorized by our local ethics committee. Patient consent was not required. Until 11/2018, we performed the TLIF procedure using the insert-and-rotate technique with placement of a banana cage at the anterior vertebral rim. From 11/2018 on, we changed our clinical practice in TLIF by placing a single straight cage obliquely across the intervertebral space. We included 41 patients who underwent a single level TLIF for L1-L5 with anterior placement of a banana cage between 11/2017 and 11/2018 and 40 patients with similar characteristics who underwent a TLIF with oblique insertion of a straight cage between 08/2018 and 03/2020. Primary outcome parameter was the change in monosegmental lordosis after fusion surgery. From the 41 patients in the banana cage group, intraoperative cage subsidence requiring a revision surgery with cage placement in a second surgery through an anterior approach occurred in three patients. Those three patients were excluded from the actual analysis of restoring SL using the banana cage. Further, we only included patients who underwent a TLIF procedure in the levels L1-5, mainly L3-4 and L4-5, since a TLIF procedure in the segment L5-S1 is fundamentally different due to an inherently greater stiffness and preoperative SL than the other segments. However, we separately analyzed and demonstrate the relordosation in the segment L5-S1 in 16 patients in the straight cage group and in 11 patients in the banana cage group. In the straight cage group, we used transforaminal insertion of two straight cages in L5-S1 in order to provide a stable base in the lumbosacral transition in the event of cranial extension of the fusion.

### 2.2 Surgical procedure

**Fig. 1:**
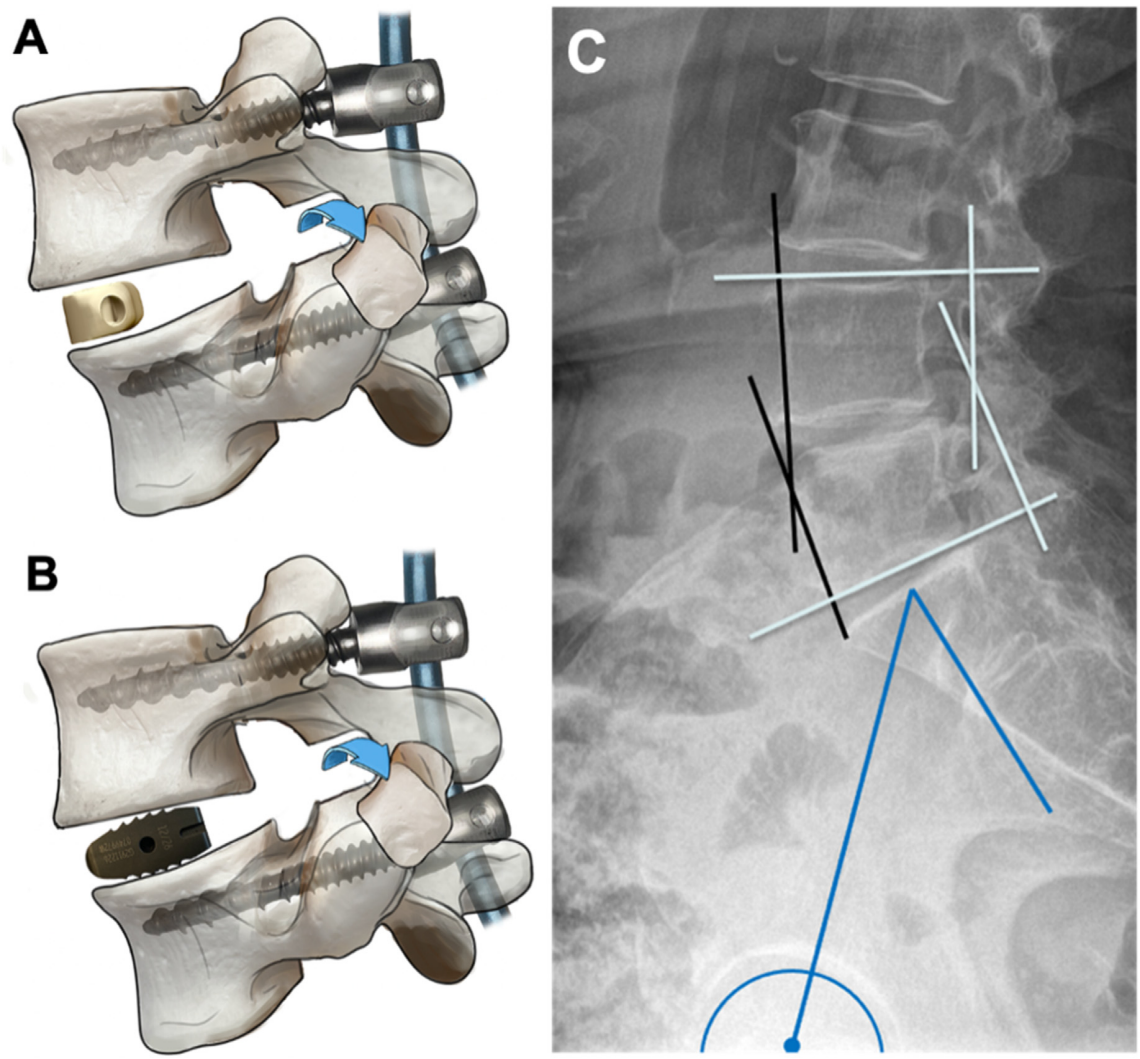
**TLIF procedure and radiographic measurement. A, B.** Dorsal release with bilateral facetectomy plus TLIF with insertion and rotation of a banana cage at the anterior vertebral edge (A) and plus TLIF with oblique insertion of a straight cage (B). **C**. Radiographic measurements. Cobb angle (white) was measured between lines paralleling the superior endplate of the upper vertebra and the inferior endplate of the lower vertebra. Anterior vertebral angle (AVB angle) (black) was measured between intersecting lines of the anterior surfaces of the vertebrae. The pelvic incidence (PI) (blue) is the angle between the perpendicular to the sacral plate at its midpoint and the line connecting the sacral plate midpoint to the center axis of the femoral heads. (For interpretation of the references to colour in this figure legend, the reader is referred to the Web version of this article.)

Patients were placed in the prone position and the level to be fused was identified using fluoroscopic guidance. An incision was made in the midline for conventional open approaches or paramedian for minimally invasive percutaneous, transmuscular pedicle screw placement and MIS tubular access. Pedicle screws were inserted with image-guidance followed by a bilateral facetectomy and (partial) laminectomy with resection of the ligamentum flavum and posterior structures under microscope visualization in order to provide an adequate dorsal release. Before the cage placement, a (sub)total discectomy was performed, and endplates were gently reamed with curettes. Then, transforaminal cage insertion was performed under distraction in order to maintain the desired disc height. In the banana cage group, a 6° lordotic semilunar cage (CRESCENT vertebral body spacer from Medtronic) was inserted and rotated at the anterior vertebral edge, whereas in the straight cage group, a straight, biconvex cage (Capstone Spinal System by Medtronic) was introduced obliquely across the intervertebral disc space. In the level L5-S1, two straight cages were placed transforaminally similar to the PLIF procedure in order to provide a stable base in the lumbosacral transition in the event of cranial extension of the fusion. Both cage types were made of titanium and packed with morselized bone. Once, the cage was placed, dorsal compression was executed via the screw heads and positions of the screws and rods were locked (Fig. 1).

### 2.3 Radiographic measurements

Preoperative and postoperative radiographic measurements of the SL were performed on plain, full spine or lumbar lateral standing x-rays. We used the Cobb angle by measuring the angle between the superior endplate of the upper vertebrae and the inferior endplate of the lower vertebrae as well as the anterior vertebral body angle (AVB angle) between intersecting lines parallel to the anterior surface of the vertebral bodies (Fig. 1) (Schuler et al., 2004). In a few cases without x-rays, CT scans were analyzed. Initial postoperative SL was assessed in all patients within one week after the surgery. For further subgroup analysis, PI was analyzed on lateral full spine or lumbar x-rays by measuring the angle between the perpendicular to the sacral plate at its midpoint and the line connecting the sacral plate midpoint to the center axis of the femoral heads. In order to study whether oblique insertion of a straight cage allows for an equal restoration of the SL in patients with a high PI, we stratified the patients according to their PI into <45°, 45°–60° and >60°

### 2.4 Bone density evaluation (HU measurement)

Patients underwent a preoperative CT scan in the Department of Radiology of the Charité – University Medicine Berlin with a mean interval ​± ​standard deviation (SD) of 13.1 ​± ​26.6 days before the surgery in the straight cage group and 6.9 ​± ​11.0 days in the banana cage group. Only unenhanced CT scans with pre-defined protocols focusing on the lumbar spine, pelvis or total spine were included. Preoperative CT examination was performed using a Canon CT Aquilion PRIME/ONE (Canon Medical Systems Corporation, Otawara, Tochigi, Japan. According to the producer, HU values are comparable between Acquilion PRIME and ONE). All Canon devices were calibrated on a regular basis to ensure accuracy and CT scans were performed following standardized parameters: 100–120 ​kVp, tube rotation time: 0.5s and scanning slice thickness: 1 ​mm. In order to assess the HU values, Phönix-PACS MERLIN Diagnostic Workcenter software was used. The type of CT window did not change the HU value and 1 ​mm slices were analyzed. CT HU values were measured by placing an oval region of interest (ROI) over an axial image of the vertebral mid-body area through the upper and lower vertebrae of the fused segment (Fig. 3). When an oval ROI was placed, Phönix-PACS MERLIN Diagnostic Workcenter software automatically calculated the mean CT HU value for this ROI. If the vertebral body was scanned at an angle, the cutting plane was aligned horizontally. No instrumented vertebrae were included. The ROI had to cover as much trabecular bone as possible. Cortical bone and heterogeneous areas, such as bone islands, posterior venous plexus and compressed bone were avoided.

### 2.5 Statistical analysis

Statistical analysis was done using GraphPad Prism 8.4.2. After analyzing the normal distribution with the D'Agostino-Pearson test, either two-tailed *t*-test or Mann-Whitney *U* test was performed to compare the difference of pre- and postoperative segmental lordosis in both groups. Probability values below 0.05 were defined as significant.

## 3 Results

### 3.1 Patient characteristics

**Fig. 2:**
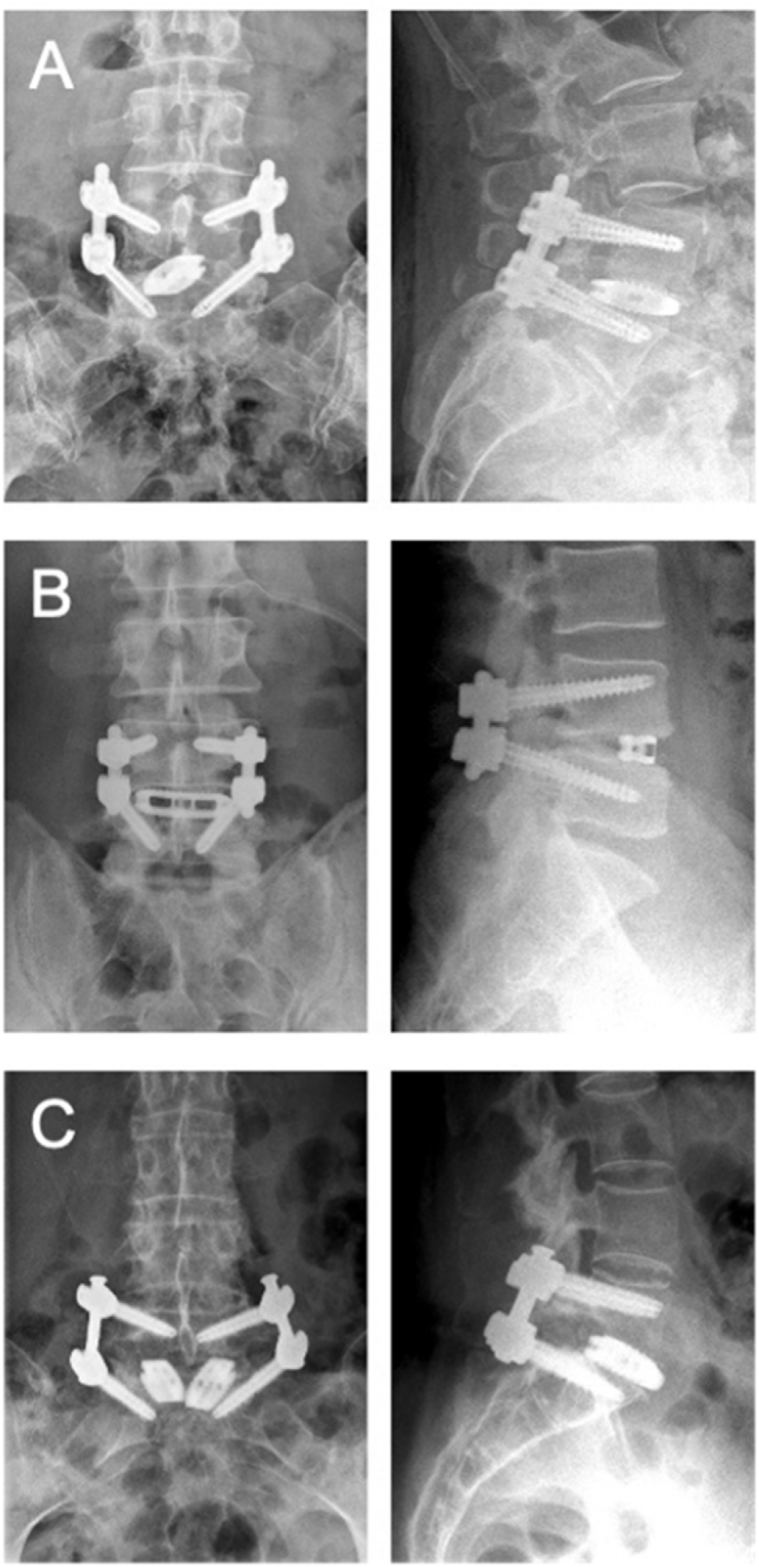
**TLIF using a straight or a banana cage.** Representative images (postoperative radiographs) of **A** TLIF with oblique insertion of a straight cage, **B** TLIF with a banana cage placed at the anterior edge and **C** TLIF using two straight cages in L5-S1.

We included 81 patients, who were operated on one level L1 to L5 between 11/2017 and 03/2020 (Table 1). For analysis of the postoperative change in SL, 40 patients were included in the straight cage group and 41 patients in the banana cage group. The mean age was 65 ​± ​11 years and in the straight cage group with 15 males (37.5%) and 25 females (62.5%) and 69 ​± ​11 years in the banana cage group with 24 males (63.2%) and 14 females (36.8%). 26 patients (65.0%) in the straight cage group and 30 patients (78.9%) in the banana cage group presented a spondylolisthesis grade I-II according to the Meyerding classification. There was no significant difference in the mean preoperative SL between the straight cage group and the banana cage group with 15.6 ​± ​9.5 and 16.8 ​± ​9.8 (Cobb angle, *t*-test, unpaired, two-tailed: p ​= ​0.57, t ​= ​0.58), respectively. We separately analyzed 16 patients with transforaminal insertion of two straight cages and 11 patients with insertion of a single banana cage in the segment L5-S1 (Fig. 2).

**Table 1:** Patient characteristics. AVB anterior vertebral body angle. HU Hounsfield Unit. MD Meyerding. SL Segmental lordosis. SD Standard deviation.

Characteristics	Banana Cage Group	Straight Cage Group	p-value
Patients	38	40	
Mean Age ± SD	69 ​± ​11	65 ​± ​11	0.15
Sex	14 w (36.8%)	25 w (62.5%)	0.04
	24 ​m (63.2%)	15 ​m (37.5%)	0.04
Spondylolisthesis MD °I-II	30 (78.9%)	26 (65.0%)	0.21
Operated Level			
L1/2	0 (0%)	1 (2.5%)	0.99
L2/3	1 (2.6%)	1 (2.5%)	0.99
L3/4	4 (10.5%)	9 (22.5%)	0.23
L4/5	33 (86.8%)	29 (72.5%)	0.16
Mean preoperative SL ± SD	Cobb 16.8 ​± ​9.8	Cobb 15.6 ​± ​9.5	0.57
	AVB 9.2 ​± ​7.7	AVB 9.4 ​± ​8.0	0.89
Mean preoperative			
HU ± SD of fused segment			
Upper vertebrae	155.0 ​± ​62.9	161.8 ​± ​48.4	0.69
Lower vetebrae	165.6 ​± ​60.0	186.5 ​± ​57.4	0.23

**Fig. 3:**
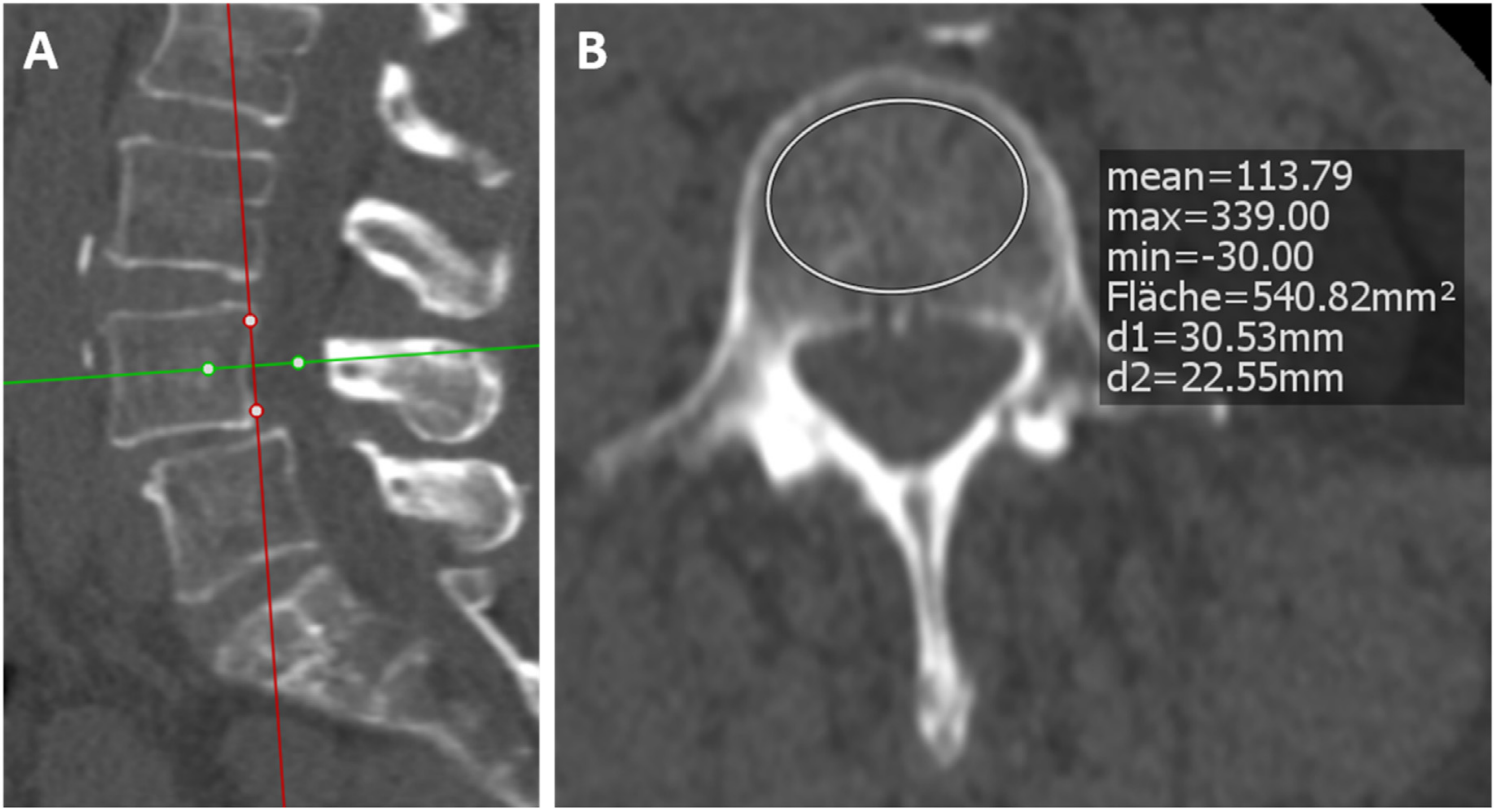
**Demonstrating HU measurement in lumbar spine CTs.** HU measurement in a lumbar spine CT scan in the **A** sagittal and **B** axial plane using an oval region of interest (ROI) over the axial image of the vertebral mid-body area. Phönix-PACS MERLIN Diagnostic Workcenter software automatically calculates the mean CT HU for the ROI in the axial plane.

### 3.2 Change in segmental lordosis

**Fig. 4:**
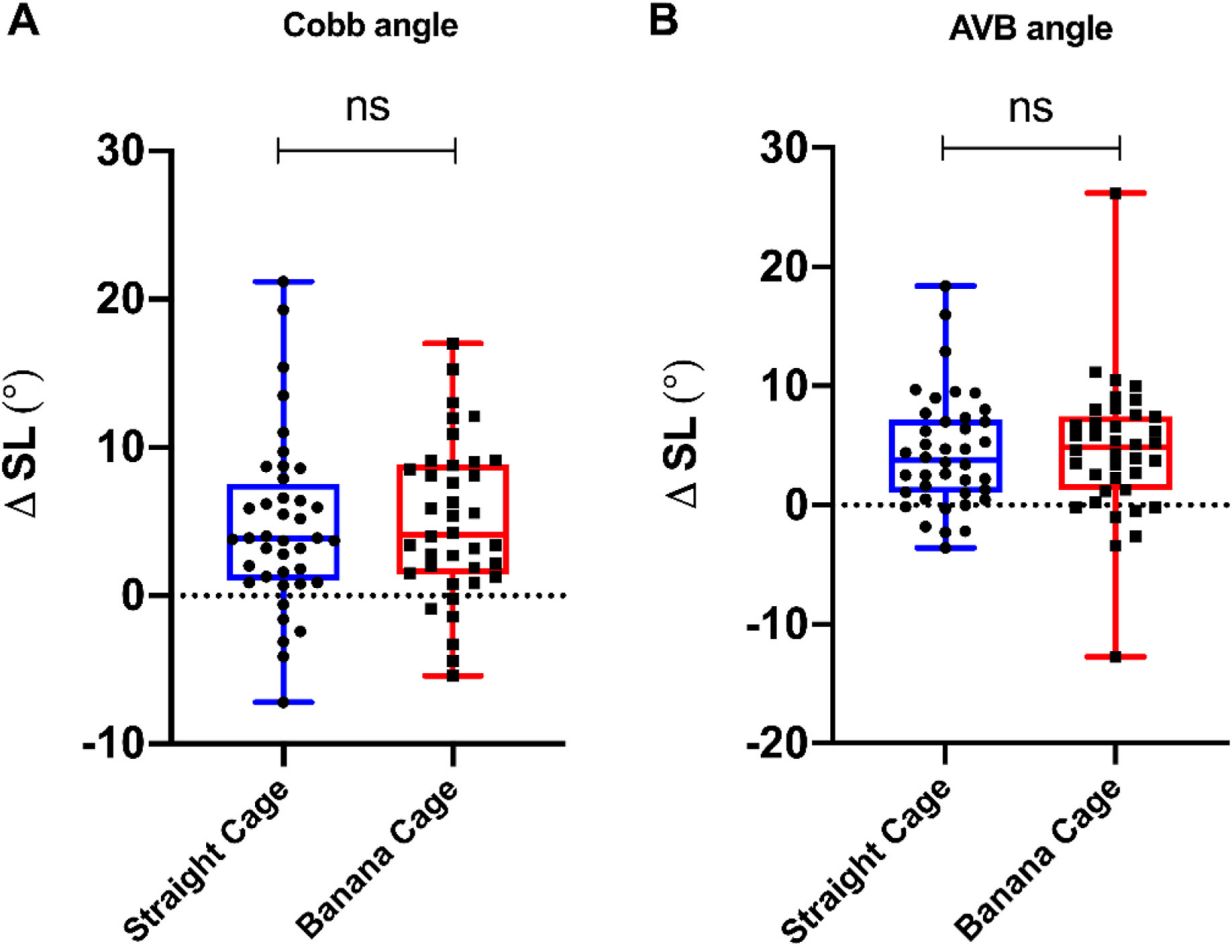
**Change in segmental lordosis.** Comparison of change in postoperative segmental lordosis of 40 patients in the straight cage group with 38 patients in the banana cage group who underwent a monosegmental TLIF in L1-5. **A** showing the Cobb angle and **B** showing the AVB angle.

In the straight cage group, SL increased from 15.6 ​± ​9.5 (Cobb angle) and 9.4 ​± ​8.0 (AVB angle) preoperatively to 20.3 ​± ​7.1 (Cobb angle) and 13.9 ​± ​5.8 (AVB angle) postoperatively. In the banana cage group, SL increased from 16.8 ​± ​9.8 (Cobb angle) and 9.2 ​± ​7.7 (AVB angle) preoperatively to 21.9 ​± ​7.7 (Cobb angle) and 13.7 ​± ​5.8 (AVB angle) postoperatively. The difference of SL in the straight cage group was 4.7 ​± ​5.8 (Cobb angle) and 4.4 ​± ​4.8 (AVB angle) compared to the difference in the banana cage group with 5.0 ​± ​5.3 (Cobb angle) and 4.6 ​± ​5.8 (AVB angle) (*t*-test, unpaired, two-tailed: p ​= ​0.82, t ​= ​0.23 Cobb angle and p ​= ​0.90, t ​= ​0.13 AVB angle) (Table 2). Thus, there was no relevant difference in the restoration of the SL between the straight cage group and the banana cage group (Fig. 4). In order to increase the stability and footprint in the lumbosacral junction we utilize transforaminal insertion of two straight cages during TLIF in the segment L5-S1. We identified 16 patients of the straight cage group who underwent a TLIF procedure in level L5-S1 with two straight cages and compared them to 11 patients of the banana group who underwent TLIF fusion of the level L5-S1. Again, there was no significant difference with restoration of the SL by 4.7° ​± ​4.5 (Cobb angle) and 3.9° ​± ​4.5 (AVB angle) in the straight cage group and by 6.1° ​± ​7.2 (Cobb angle) and 5.2° ​± ​5.3 (AVB angle) in the banana cage group (*t*-test, unpaired, two-tailed: p ​= ​0.54, t ​= ​0.62 Cobb angle and p ​= ​0.49, t ​= ​0.71 AVB angle) (Table 2).

**Table 2:** Postoperative change in segmental lordosis. Comparison of preoperative and postoperative segmental lordosis of 40 patients in the straight cage group with 38 patients in the banana cage group (left) and comparison of preoperative and postoperative segmental lordosis of 16 patients with transforaminal insertion of two straight cages in L5-S1 with 11 patients with a banana cage in L5-S1 (right). In each cell, the upper value gives the Cobb angle and the lower one the anterior vertebral body angle (AVB angle). SD Standard deviation; SL segmental lordosis; ΔSL SL postoperative - SL preoperative.

TLIF construct	Straight Cage L1-L5	Banana Cage L1-L5	p-value	Double Straight Cage L5-S1	Single Banana Cage L5-S1	p-value
Mean SL	15.6 ​± ​9.5	16.8 ​± ​9.8	0.69	17.8 ​± ​6.2	17.6 ​± ​10.1	0.54
preop ± SD	9.4 ​± ​8.0	9.2 ​± ​7.7	0.89	46.1 ​± ​9.9	45.2 ​± ​10.4	0.84
Mean SL	20.3 ​± ​7.1	21.9 ​± ​7.7	0.36	22.5 ​± ​5.8	23.7 ​± ​8.3	0.65
postop ± SD	13.9 ​± ​5.8	13.7 ​± ​5.8	0.89	50.0 ​± ​8.6	50.5 ​± ​8.3	0.88
Δ SL ± SD	4.7 ​± ​5.8	5.0 ​± ​5.3	0.82	4.7 ​± ​4.5	6.1 ​± ​7.2	0.54
	4.4 ​± ​4.8	4.6 ​± ​5.8	0.90	3.9 ​± ​4.5	5.2 ​± ​5.3	0.49
n patients	40	38		16	11

### 3.3 Relevance of high pelvic incidence

Since the amount of LL is directly controlled by and proportional to the PI, a high PI may necessitate a higher LL and should be taken into account during appropriate preoperative planning (Ames et al., 2012; Mehta et al., 2015; Smith et al., 2013). Therefore, in order to study whether oblique insertion of a straight cage allows for an equal restoration of the SL in patients with a high PI, we stratified the patients according to their PI into <45°, 45°–60° and >60° (Table 3). Compared to the use of a banana cage, oblique insertion of a straight cage demonstrated to enable an equally effective postoperative correction of the SL in patients with a high PI ​> ​60°. In the fused segment, SL increased by 6.1 ​± ​6.5 (Cobb) and 5.5 ​± ​5.1 (AVB angle) in the straight cage group compared to 3.8 ​± ​5.7 (Cobb angle) and 1.1 ​± ​5.8 (AVB angle) in the banana cage group (*t*-test, unpaired, two-tailed: p ​= ​0.33, t ​= ​1.0 Cobb angle and p ​= ​0.04, t ​= ​2.2 AVB angle).

**Table 3:** Comparison of preoperative and postoperative segmental lordosis in subgroups according to the patients' pelvic incidence. In each cell, the upper value demonstrates the Cobb angle and the lower one demonstrates the anterior vertebral body angle (AVB angle). SD Standard deviation; SL segmental lordosis; ΔSL SL postoperative - SL preoperative.

TLIF construct	Oblique Cage PI ​> ​60°	Banana Cage PI ​> ​60°	p-value	Oblique Cage PI 45–60°	Banana Cage PI 45–60°	p-value	Oblique Cage PI ​< ​45°	Banana Cage PI ​< ​45°	p-value
Mean PI ± SD	68.3 ​± ​8.1	69.2 ​± ​6.5	0.70	52.6 ​± ​5.2	53.3 ​± ​3.6	0.68	36.9 ​± ​4.4	39.7 ​± ​4.0	0.18
Mean SL	18.5 ​± ​11.8	21.1 ​± ​8.7	0.60	14.7 ​± ​6.3	15.0 ​± ​10.6	0.91	10.2 ​± ​5.9	15.2 ​± ​9.0	0.29
preop ± SD	11.0 ​± ​9.5	13.6 ​± ​7.8	0.45	8.5 ​± ​7.3	7.5 ​± ​7.3	0.70	7.2 ​± ​4.4	7.1 ​± ​6.8	0.72
Mean SL	24.6 ​± ​7.3	24.8 ​± ​7.5	0.43	17.7 ​± ​4.7	20.7 ​± ​8.6	0.25	14.8 ​± ​3.9	20.6 ​± ​6.3	0.05
postop ± SD	16.7 ​± ​5.5	14.8 ​± ​5.7	0.36	11.8 ​± ​5.8	13.9 ​± ​6.4	0.35	11.2 ​± ​3.2	12.5 ​± ​5.3	0.77
Δ SL ± SD	6.1 ​± ​6.5	3.8 ​± ​5.7	0.33	3.1 ​± ​5.9	5.7 ​± ​5.0	0.19	4.6 ​± ​2.8	5.4 ​± ​5.7	0.75
	5.5 ​± ​5.1	1.1 ​± ​5.8	0.04	3.3 ​± ​5.0	6.4 ​± ​6.4	0.14	4.0 ​± ​3.3	5.4 ​± ​3.4	0.40
n patients	18	11		15	16		7	11	

### 3.4 Endplate violation during cage placement

Oblique insertion of the straight cage does not require repositioning after cage placement. Therefore, we postulated that oblique insertion of the straight cage does not only prove to be equally effective in restoring the SL but also causes less intraoperative endplate damage and cage subsidence compared to the insertion and rotation of the banana cage. In the banana cage group, in 3 out of 41 patients (7.3%), rotation of the banana cage caused intraoperative endplate damage and cage subsidence. Here, only dorsal instrumentation was performed during the primary surgery and implantation of the cage was done via an anterior approach during a second surgery. Only the other 38 patients, who underwent a successful TLIF procedure using a banana cage, were included for further analysis of the SL. In the straight cage group in contrast, no intraoperative cage subsidence requiring a revision surgery occurred.

### 3.5 Assessing bone density using HU values

The risk of intraoperative cage subsidence is highly influenced by the patient's bone quality. Thus, we used the HU values of preoperative spinal CT scans in order to compare the bone density in both groups. The mean HU values with SD for the upper vertebral bodies of the fused segment were 161.8 ​± ​48,3 in the straight cage group and 155.0 ​± ​62.9 in the banana cage group (Table 1) (*t*-test, unpaired, two-tailed: p ​= ​0.69, t ​= ​0.40). The mean HU values with SD for the lower vertebral bodies of the fused segment were 186.5 ​± ​57.4 in the straight cage group and 165.6 ​± ​60.0 in the banana cage group (Table 1) (*t*-test, unpaired, two-tailed: p ​= ​0.23, t ​= ​1.2). In the literature, HU values differ between each vertebral body of the lumbar spine (Schreiber et al., 2011; Zou et al., 2019). However, in our study, we mainly included patients who were operated on the segments L3/4 and mostly L4/5, limiting the bias of different HU thresholds. We found that there was no significant difference in the HU values of the upper and lower vertebral bodies of the fused segment between the straight cage group and the banana cage group.

## 4 Discussion

The principal novel finding of our study is, that in terms of restoring the SL, oblique insertion of a straight cage across the intervertebral disc space in one-level TLIF procedure is non-inferior to the insert-and-rotate technique with placement of a conventional banana-shaped cage at the anterior vertebral edge. However, the banana cage showed a higher rate of intraoperative cage subsidence requiring revision surgery. These findings contradict the current idea that placing the interbody cage as anteriorly as possible not only provides biomechanical advantages but also allows for the most effective and safest restoration of the SL.

### 4.1 Potential restoring the SL

With increasing understanding of the relevance of the pelvic position and the spinopelvic alignment, restoring SL and LL have increasingly become important parameters in the context of lumbar spinal fusion surgery (Schwab et al., 2013). The LL is proportional to the PI and the PI-LL mismatch assesses the loss of LL in relation to the pelvic morphology (Schwab et al., 2013; Lafage et al., 2016). PI-LL is also closely correlated with the PT, since reduced LL leads to a compensatory retroversion of the pelvis reflected in an increased PT (Schwab et al., 2012, 2013). In a regression model, Schwab et al. even suggested a PT of 22° or more and a PI−LL mismatch of 11° or more as guideline threshold parameters for preoperative planning since increased PT and PI-LL mismatch demonstrated a strong correlation with worsening HRQOL scores predictive of higher scores on the ODI (Schwab et al., 2002, 2013; Lafage et al., 2009). However, the only variable that can be directly influenced in a lumbar spinal fusion surgery is the LL. Therefore, multiple formulas have been proposed for preoperative calculation of the optimum LL necessary to reduce PT and to restore a balanced spinopelvic alignment with a suitable inclination of the pelvis (Lafage et al., 2011, 2012; Kim et al., 2006). Further, recent studies revealed a significant implication of the PI-LL mismatch on the risk assessment of postoperative failure and development of ALD after spinal lumbar fusion surgery (Schwab et al., 2013; Rothenfluh et al., 2015; Aoki et al., 2015; Tempel et al., 2017) since patients presenting a high spinopelvic mismatch exhibit higher shear stress of the adjacent segment before and after surgery (Senteler et al., 2014). In fact, Tempel et al. demonstrated, that a PI-LL mismatch of >11° had a predictive value of 75% for developing ALD after single-level TLIF requiring surgical revision (Tempel et al., 2017). This highlights the increasing importance of restoring the SL during the TLIF procedure. However, the influence of the cage design and positioning during lumbar spinal fusion surgery still remains controversial (Choi et al., 2018; Cho et al., 2008; Tassemeier et al., 2018).

Currently, the widely preferred strategy implies the usage of curved banana cages, which are supposed to offer a higher potential in restoring the disc height and postoperative SL (Rice et al., 2016; Tassemeier et al., 2018). However, in our study, both the banana cage as well as the straight cage were equally effective in restoring the postoperative SL, contradicting the currently accepted opinion. Here, the banana cage and the straight cage showed a similar postoperative gain in SL of 5.0 ​± ​5.3 and 4.7 ​± ​5.8, respectively (Table 2). The achieved relordosation correlates with some higher values reported in the literature, like Tye et al. with 5.7° and Berlin et al. with 4.7° relordosation (Tye et al., 2016; Berlin et al., 2021). But in some studies, less preservation or even decrease of the SL after TLIF was reported (Rice et al., 2016; Hsieh et al., 2007; Vaishnav et al., 2020). In those studies, the authors used a unilateral approach with unilateral facetectomy. In our study, we utilized a bilateral facetectomy with partial or complete laminectomy. The similar rates of postoperative correction of the SL using both the straight cage and the banana cage implicate that the choice of cage design is actually less crucial than the surgical technique. In fact, an extended dorsal release is the key factor, if the surgeon intends to achieve a greater postoperative SL and may include a Smith-Peterson osteotomy or an osteotomy type 2 according to Schwab with bilateral facetectomy and partial or complete laminectomy. The dorsal release mainly determines the extent of possible correction and allows to insert the desired cage size under distraction followed by an adequate dorsal compression without bony obstruction in the posterior aspect (Schwab et al., 2015; Snyder et al., 2019; Robertson et al., 2018b). A biomechanical study by Snyder et al. demonstrated a significantly greater change in foraminal height with better dorsal compression after a complete bilateral facetectomy, which resulted in a greater final lordosis angle of the fused segment compared to a unilateral facetectomy (Snyder et al., 2019). A bilateral facetectomy also allows for a significantly increased restoration of the postoperative disc space height (Archer et al., 2020). The preoperative loss of the disc space height might be another important variable that determines the possible degree of restoring SL regardless of the cage type. Thus, the same correction of SL in our study using both the straight and the banana cage suggests, that in the setting of an adequate dorsal release and dorsal compression, both cage types might effectively restore the disc height and support restoration of the SL. If a spinal deformity needs higher correction, lordotic cages with greater lordotic angles like 15° or more demonstrate a better restoration of the SL (Hong et al., 2017). Here, we used a banana cage with inherent 6° lordosis while the straight cage did not have a lordotic angle but an asymmetric biconvex shape. Even greater correction can be achieved using an OLIF or ALIF approach in the lower segments (Hsieh et al., 2007).

### 4.2 Intraoperative cage subsidence

Anterior placement of the banana cage is not only supposed to allow for a better restoration of the SL, it is also supposed to reduce the risk of cage subsidence (Yao et al., 2020). However, the biconvex, elliptical design of the straight cage might offer improved biomechanical properties (von der Hoeh et al., 2018; Chen et al., 2005). A prospective randomized trial from Choi et al. demonstrated a significantly reduced long-term incidence of 17.5% cage subsidence after 12 months in the straight cage group compared to 31.8% in the banana cage group (Choi et al., 2018). This is supported by recent biomechanical studies showing that insertion of a straight cage with an adequate length provides biapophyseal footing, transferring the axial forces to the stronger apophyseal ring (von der Hoeh et al., 2018). But there is little report in the literature about the influence of the cage design on immediate, intraoperative cage subsidence.

In our study, three patients in the banana cage group (7.3%), but none in the straight cage group, had to undergo a revision surgery after levering and rotating of the banana cage caused intraoperative endplate damage with consequent cage subsidence. In those patients, only dorsal instrumentation was performed in the primary surgery with inserting the cage through an OLIF approach within a second surgery. Intraoperative cage subsidence depends on multiple surgical aspects and might include aggressive endplate reaming, the surgeon's experience, choosing an oversized cage or, in case of the banana cage, misplacement of the implant in the weaker central part of the vertebral body instead at the anterior vertebral rim (Faundez et al., 2008; Yao et al., 2020). However, despite technical aspects, patient specific characteristics exert a significant influence on the risk of intraoperative cage subsidence as well. While the procedures in the three patients, who had to undergo a revision due to intraoperative cage subsidence, were performed by our most experienced spine surgeons, who had been using the banana cage for years, assessing the vertebral bone density using HU values revealed a reduced bone quality in all of the three patients. The HU values of the upper and of the lower vertebral bodies of the fused segment were 70.1 and 80.1 in the first, 81.7 and 88.4 in the second and 90.7 and 99.1 in the third patient, respectively. All three patients were operated on the segment L4/5 and in this segment, a HU value around 100 can be correlated with osteopenic (T-score less than −1.0 or greater than −2.5 according to the WHO classification) and a HU value around 80 with osteoporotic bone density (T-score −2.5 or less) (Schreiber et al., 2011; Zou et al., 2019). Regarding the mean HU values of both groups, there was no significant difference. Thus, the need for repositioning after insertion of the banana cage seems to be an additional risk factor that has to be considered especially in patients with a reduced bone quality. In those patients, aggressive endplate reaming and rotating of the banana cage might entail a higher risk for endplate violation and intraoperative cage subsidence (Formby et al., 2016; Yao et al., 2020). However, the straight cage does not require repositioning after insertion and therefore, may reduce the risk for intraoperative cage subsidence in patients with a reduced bone density.

### 4.3 Limitations

Our study had a number of limitations. First, we demonstrated an equally effective restoration of the immediate postoperative SL with both cage types. However, long-term follow-up is needed to evaluate the maintenance of the postoperatively gained SL as well as the different fusion rate between the two cage types. Second, bigger patient samples will be needed in order to confirm the higher risk for intraoperative cage subsidence in the banana cage group. Our data suggest a reduced risk for intraoperative cage subsidence in patients with reduced bone quality using the straight cage, however, we could only report three patients requiring revision surgery due to intraoperative cage subsidence in the banana cage group. Third, there is no data regarding the clinical outcome in the two groups. And fourth, in a few patients without preoperative plain full spine or lumbar lateral standing x-rays, CT scans were analyzed, and the real lordosis may be significantly different from standing to supine. However, the lack of a preoperative standing x-ray in 100% of the patients applies to both groups and is balanced between the groups. Thus this bias should not affect the message of the study.

## 5 Conclusions

Our data suggest that an effective restoration of the SL during the TLIF procedure mainly depends on surgical aspects. Provided an adequate dorsal release, both cage types seem to support an equally effective restoration of the SL. However, since the insertion of the straight is technically easier and might reduce the risk for intraoperative cage subsidence in patients with a reduced bone density, we changed our clinical practice in TLIF procedure by using the oblique insertion of the straight cage across the intervertebral disc space.

## Disclosures

We have no conflicts of interest to disclose. There was no financial support or industry affiliations. All authors have approved the final article. This research did not receive any specific grant from funding agencies in the public, commercial, or not-for-profit sectors.

## Declaration of competing interests

The authors declare that they have no known competing financial interests or personal relationships that could have appeared to influence the work reported in this paper.
